# Supplementary material for: Between Stress and Response: Function and Localization of Mechanosensitive Ca2+ Channels in Herbaceous and Perennial Plants
Source: Int J Mol Sci. 2021 Oct 13;22(20):11043. doi: 10.3390/ijms222011043 (PMC8538497; doi:10.3390/ijms222011043)
Supplement: Supplementary file 1 [file ijms-22-11043-s001.zip › ijms-1402284-supplementary.pdf]

Species	Gene name	Accession number	Sequence Source
Arabidopsis thaliana	AtOSCA1.1	AT4G04340.1	TAIR10, Phytozome
	AtOSCA1.2	AT4G22120.1	
	AtOSCA1.3	AT1G11960.1	
	AtOSCA1.4	AT1G62320.1	
	AtOSCA1.5	AT3G21620.1	
	AtOSCA1.6	AT4G15430.1	
	AtOSCA1.7	AT4G02900.1	
	AtOSCA1.8	AT1G32090.1	
	AtOSCA2.1	AT1G58520.1	
	AtOSCA2.2	AT1G10090.1	
	AtOSCA2.3	AT3G01100.1	
	AtOSCA2.4	AT1G69450.1	
	AtOSCA2.5	AT3G54510.1	
	AtOSCA3.1	AT1G30360.1	
	AtOSCA4.1	AT4G35870.1	
	AtMCA1	AT4G35920.1	
	AtMCA2	AT2G17780.1	
	AtPiezo1	AT2G48060.1	
Populus trichocarpa	PtOSCA1.1	Potri.004G005900	V3.1, Phytozome
	PtOSCA1.2	Potri.004G006000	
	PtOSCA1.3	Potri.011G009900	
	PtOSCA1.4	Potri.004G006100	
	PtOSCA1.5	Potri.011G009800	
	PtOSCA1.6	Potri.002G226800	
	PtOSCA1.7	Potri.014G156100	
	PtOSCA1.8	Potri.001G133800	
	PtOSCA1.9	Potri.003G099800	
	PtOSCA2.1	Potri.003G200900	
	PtOSCA2.2	Potri.002G113800	
	PtOSCA2.3	Potri.004G115800	
	PtOSCA2.4	Potri.008G091200	
	PtOSCA2.5	Potri.010G164100	
	PtOSCA3.1	Potri.001G358300	
	PtOSCA4.1	Potri.005G108600	
	PtOSCA4.2	Potri.007G063700	
	PtMCA1	Potri.005G110000	
	PtMCA2	Potri.010G184700	
	PtPiezo1	Potri.014G137200	
	PtPiezo2	Potri.005G037700	
